# The SIMPLER Nutrition Pathway for Fragility Fractures: A Quality Improvement Initiative

**DOI:** 10.3390/nu17121987

**Published:** 2025-06-12

**Authors:** Jack J. Bell, Olof Gudny Geirsdottir, Antony Johansen, Julie Santy-Tomlinson, Frede Frihagen, Rhona McGlasson, Emma Sutton, Karen Hertz

**Affiliations:** 1 Faculty of Food Science and Nutrition, School of Health at the University of Iceland, 102 Reykjavik, Iceland; 2 Fragility Fracture Network, 4053 Basel, Switzerland; 3 School of Medicine, University Hospital of Wales, Cardiff University, Cardiff CF14 4XW, UK; 4 Department of Orthopaedic Surgery, Østfold Hospital Trust, 1712 Grålum, Norway; 5 Institute of Clinical Medicine, University of Oslo, 0407 Oslo, Norway; 6 Department of Nursing and Midwifery, School of Health Sciences, College of Medicine and Health, University of Birmingham, Edgbaston, Birmingham B15 2TT, UK; 7 Royal Stoke University Hospital, University Hospitals of North Midlands, Stoke-on-Trent ST4 6QG, UK

**Keywords:** hip fracture, fragility fracture, nutritional support, malnutrition, implementation, hospitals, improvement, quality improvement, protocol

## Abstract

**Background/Objectives:** Malnutrition is a key contributor to poor outcomes in older adults with fragility fractures, increasing the risk of complications, functional decline, prolonged hospital stays, mortality, and healthcare costs. Substantial evidence limited to hip fracture supports early, interdisciplinary nutrition care. However, global audits reveal that most hip fracture patients do not receive recommended interventions. This quality improvement (QI) project aimed to co-create and test a pathway and toolkit to help apply evidence-based nutrition care in different fragility fracture settings globally. **Methods:** The SIMPLER Pathway and toolkit (SIMPLER) were developed through a multiphase, co-creation QI initiative (2018–2025), guided by the Knowledge-to-Action framework. Global experts and clinical teams synthesized evidence, identified the “know-do” gap, and adapted SIMPLER to context through iterative action–reflection cycles. The Model for Improvement guided team building, goal setting, testing changes, and measuring outcomes at pilot sites. **Results:** Over 100 co-creation activities between 2018 and 2025 engaged staff and patients to shape and refine SIMPLER. A global clinician survey (n = 308, 46 countries), two bi-national audits (n = 965, 63 hospitals), and qualitative interviews (n = 15) confirmed a widespread evidence-practice gap. The pathway and toolkit were pilot-tested in five hospitals across four countries, with action–reflection cycles enabling continuous refinement of prioritized nutrition improvements tailored to the local context. Following endorsement in late 2024, 46 healthcare services in 23 countries have formally committed to implementing SIMPLER. **Conclusions:** The SIMPLER Nutrition Pathway provides a scalable, adaptable framework to support the delivery of evidence-based nutrition care in fragility fracture settings. A global evaluation is underway.

## 1. Introduction

### Background

Fragility fractures are fractures resulting from low-energy trauma—mechanical forces that would not normally lead to a fracture, such as a fall from standing height or less. In 2019, the burden of disability associated with fractures reached 25.8 million years lived with disability (YLDs), marking a 65% increase since 1990 [1]. The most affected sites are the spine, hip, distal forearm, and proximal humerus. The World Health Organization identifies hip and vertebral fractures as the most serious types of fragility fractures, given their strong association with increased morbidity, mortality, and healthcare burden [1].

Hip fracture patients are often identified as the most nutritionally vulnerable group within the fragility fracture population and among older adults more generally [2,3]. Consequently, most nutrition-focused research, guidelines, and care standards in fragility fracture are centred on hip fracture [4,5,6,7,8,9]. As a result, hip fracture services often serve as the main focus for nutrition research and improvement works, with the aim of translating findings to all fragility fracture care over time.

Up to one-half of hip fracture patients are malnourished on admission and without timely intervention; single-site research suggests this may increase to two thirds of inpatients by the time of discharge [8,9,10,11]. Malnutrition is a strong predictor of poor outcomes for people who experience hip fractures including delayed mobility, complications, increased length of stay, 12-month mortality, and higher healthcare costs [2,12,13]. This association appears independent of body mass index [14].

Most nutrition screening tools have poor sensitivity in hip fracture inpatients. This can lead to the underdiagnosis and undertreatment of malnutrition [8,9,15,16,17,18]. Consequently, all hip fracture patients should receive interdisciplinary, multicomponent care until they are assessed as well nourished, clinically stable, no longer at risk of nutritional deterioration, and meeting their post-operative, post-trauma nutritional requirements [8,11,17,18,19,20]. Multicomponent nutrition care actions commonly include avoiding prolonged fasting, offering high quality, nutrient-dense food choices, oral nutritional supplements, tailored nutrition information for patients and carers, and clinical handover to ongoing care providers if malnutrition or risk of malnutrition are not resolved at discharge [17,21,22,23,24]. However, across global regions, early, interdisciplinary, multicomponent nutrition care is not routinely provided to older adults with hip fractures [11,25,26].

This manuscript outlines the background and rationale for the SIMPLER Nutrition Pathway and Toolkit. It also describes the co-creation methods used in its development and field testing. The results describe the quality improvement methodology, stakeholder engagement strategies, and implementation processes used during the co-creation phase. They also introduce the final version of the SIMPLER Nutrition Pathway for Fragility Fractures, now presented in the peer-reviewed literature for global adoption. Effectiveness outcomes, including patient-level impacts, are being reported separately.

## 2. Materials and Methods

The SIMPLER Nutrition Pathway for Fragility Fractures and associated toolkit resources were co-created and field-tested as a non-linear, multiphase quality improvement initiative. The implementation approach was informed by the Knowledge to Action (KTA) framework, with quality improvement guided by the Model for Improvement and principles of co-creation [27,28,29].

### 2.1. KTA Implementation Approach

The KTA framework was chosen as a widely used implementation process model. This model integrates a **knowledge creation cycle** with an iterative **action cycle** that helps users translate evidence into practice.

#### 2.1.1. The KTA Knowledge Creation Cycle

The ***knowledge creation cycle*** refines evidence through synthesis, product development, and product tailoring for end-users. To create SIMPLER, multiple knowledge creation cycles applied a structured global database, grey literature, and relevant organizational website searches to identify knowledge sources. Knowledge from these primary studies, systematic, non-systematic, scoping and narrative reviews, and meta-analyses then informed the development and creation of the final knowledge tool for actioning, that is SIMPLER.

#### 2.1.2. The KTA Action Cycle

The ***action cycle*** guides the systematic implementation, adaptation, and sustainability of knowledge in real-world settings. It also supports continuous refinement and stakeholder engagement to translate knowledge into sustainable, scalable practice improvements. Multiple action cycle phases were applied to implement, and iteratively refine SIMPLER into practice.

The *Identify Problem/Determine the Know–Do Gap phase* targeted the identification of the gap between nutrition care recommendations and real-world practice. This phase applied patient, clinician, and facility level audits, surveys, interviews, focus groups, workshops, and expert question and answer panels at the hospital, national, global region, and global levels.

In the *Identify, Review, Select Knowledge phase*, key evidence sources from the knowledge creation cycle were refined and tailored to develop the initial SIMPLER pathway and implementation approach. Consensus approval from the FFN (Fragility Fracture Network www.fragilityfracturenetwork.org (accessed on 10 May 2025)) SIMPLER Implementation Committee and pilot sites to these were then obtained before pilot implementation. Pilot sites then applied *Adapt Knowledge to Local Context* phases with support from an implementation specialist, the FFN Implementation Committee and Site Network members to *select, tailor, and implement SIMPLER* into their local settings. This included processes to *Assess Barriers and Enablers to Knowledge [SIMPLER] Use*, *Monitor Knowledge Use, and Evaluate Outcomes phases.* These phases applied mixed methods approaches to data collection including audits, patient-reported measures, semi-structured qualitative interviews, site support meetings, surveys, webinars, and workshops.

The final co-creation phase focused on *Sustaining Knowledge Use.* The endorsement was obtained from all pilot sites at a face-to-face meeting in Istanbul, Türkiye in October 2024. An expression of interest for new adopter sites to implement SIMPLER was distributed using a snowball approach. A flyer with a link to an electronic form requesting a formal expression of interest was circulated to FFN members and promoted at the FFN Regional Congresses and at the Australian and New Zealand Hip Fracture Registry ‘HipFest’.

### 2.2. Quality Improvement Approach

The Model for Improvement [28,29] was selected for its structured, iterative approach to guiding the SIMPLER healthcare improvement process at pilot sites. Like the KTA, this model was chosen as it actively solicits stakeholder engagement, data-driven decision-making, and continuous learning to implement measurable and sustainable quality improvement actions that can be tailored to diverse settings. Teams were supported by an expert facilitator and toolkit resources to apply the following key improvement steps during piloting: forming a team (or teams), setting aims, establishing measures, selecting changes, implementing changes, and sustaining and spreading improvements. Processes for establishing measures and identifying changes applied small-scale, test–retest Plan–Do–Study–Act (PDSA) cycles prior to full implementation.

### 2.3. Co-Creation and Co-Production Approach

Across global contexts, the terms co-creation, co-production, and co-design are often used interchangeably, with overlapping definitions. However, all acknowledge the complex relationships between academics, clinicians, patients, researchers, and other stakeholders working together to generate and adapt knowledge, develop ideas, and drive improvement. This quality improvement program primarily followed principles associated with co-creation and co-production; for simplicity, the term “co-creation” is used throughout the manuscript. Co-creation is the collaborative development of knowledge, tools, or solutions through partnerships between stakeholders—including academics, patients, clinicians, policymakers, and field experts [30]. Co-production explores the role of patients as active contributors in a shared process (e.g., shared decision-making) and focuses on how best to support their interactions with healthcare staff and systems [31]. In line with Greenhalgh et al., we placed individual experiences at the heart of the co-creation process. Academic researchers were engaged as ‘knowledge brokers’ and together with implementation facilitators, linking knowledge generators with change champions and end users to facilitate translation of evidence into clinical practice [32,33]. Levels of patient and care partner engagement varied considerably across the diverse range of sites and improvement actions [34].

## 3. Results

These results describe the co-creation and pilot implementation process for the SIMPLER Nutrition Pathway, including rationale, development, and field testing, and introduce the Pathway and Toolkit. Effectiveness outcomes are being reported separately.

Across the course of implementation, processes were overseen by the FFN Education Committee Nutrition Advisory Board, the Global SIMPLER Implementation Steering Committee, SIMPLER Pilot Site Champions, and local site teams. More than 100 targeted co-creation engagement processes were undertaken between 2018 and 2024 across FFN groups in Asia-Pacific, Europe, and the Americas (Table 1).

### 3.1. Knowledge Creation Cycle

Key knowledge sources identified during the knowledge creation cycle are referenced throughout this manuscript. A scoping review and narrative review will be published separately.

### 3.2. Knowledge-to-Action Cycle

A key focus of the *Identify Problem/Determine the Know–Do Gap phase* cycles was to pinpoint the discrepancy between care recommendations and real-world practice across global regions. Findings from two bi-national patient-level and facility-level audits (n = 965 patients; 63 participating hospitals) were triangulated by a global survey of 308 clinicians from 46 countries to confirm that nutrition guidelines were routinely unmet [35,36,37,38]. These quantitative findings were corroborated by numerous other data sources, including qualitative interviews with 15 interprofessional leaders in the field, pilot site participants, and two global interprofessional workshops conducted in 2018 and 2024 (estimated combined attendance of 300 participants). This confirmed a substantial and clinically significant evidence-to-practice gap and the need for change.

The *Identify/Review/Select Knowledge* phase synthesized key knowledge sources to create the initial SIMPLER pathway, protocol, and toolkit resources. This draft received full consensus approval for pilot implementation from the FFN SIMPLER Implementation and Site Network committees in early 2024 [39,40].

Following a call for expressions of interest to FFN members, five hospitals across four countries were selected as pilot sites after establishing their local teams and setting aims. Throughout 2024, pilot sites participated in email communications, face-to-face and online meetings, workshops, and focus groups to refine the SIMPLER pathway, protocol, and toolkit resources. Sites were supported by structured engagement and implementation processes (Table 1), including regular meetings with an implementation specialist experienced in nutrition care improvements across international settings. This approach facilitated continuous, iterative site-led adaption to the local context while simultaneously informing improvements to the pathway, protocol, and toolkit resources.

The final phase of implementation, *Sustaining Knowledge Use* focused on approval of the final versions for sustained and scaled-up knowledge use. A focus group was convened at the FFN meeting in Istanbul, Türkiye, bringing together representatives from pilot sites and co-leads of the Implementation Steering Committee. A unanimous consensus was reached on the final pathway. Focus group members noted that, while it offered structured guidance and core examples, implementing sites would need to adapt and co-create the pathway, protocol, and toolkit to suit their local contexts. Following the circulation of an expression of interest for new SIMPLER sites, 46 healthcare services from 23 countries across 5 of the 6 World Health Organization global regions [41] had committed, at the time of writing, to implementing and evaluating SIMPLER in their settings.

### 3.3. Introducing SIMPLER

**Figure 1:**
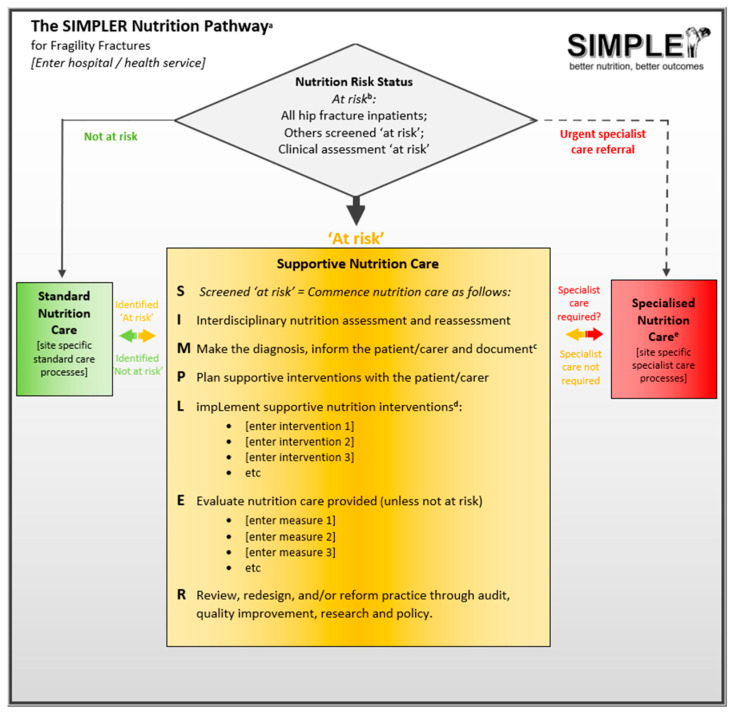
The SIMPLER Nutrition Pathway for Fragility Fractures. ^a^ The SIMPLER Pathway for inpatients with hip fracture is applied and adapted with permission from Queensland Health [22,48] and is provided here for educational purposes only. It must be customized to local contexts and governance requirements prior to implementation. ^b^ Most screening tools have demonstrated poor sensitivity in hip fracture inpatients; this can result in underdiagnosis and undertreatment of malnutrition. Consider routinely treating all hip fracture patients at risk of malnutrition until diagnosed as malnourished or not [8,9,15,16,17,18]. For other fragility fracture patients, consider using a validated screening tool such as the Mini-Nutritional Assessment Short Form [49], Malnutrition Screening Tool [50], or Malnutrition Universal Screening Tool [45]. Other high-risk conditions, for example, delirium may be considered by treating teams as ‘at risk’ until assessed otherwise. ^c^ Using a validated diagnostic tool for example, the Global Leadership Initiative on Malnutrition (GLIM) criteria [51], International Classification of Diseases (ICD) criteria [52], the Subjective Global Assessment [53], the Mini-Nutritional Assessment Short Form (score < 8) [49,54]. This may be performed after supportive interventions are commenced. ^d^ Within 72 h (target < 24 h) of presentation with a hip fracture. Highly recommended ‘core’ interventions are detailed in the toolkit as the ‘SIMPLER Six’. For detailed examples of additional SIMPLE(R) supportive nutrition care interventions, consider the Multimodal, multidisciplinary nutritional care in acute hip fracture model [11], Orthogeriatrics Nutrition care chapter [55], Interdisciplinary Nutritional Management and Care for Older Adults Textbook [23], The SIMPLE Approach systematized, interdisciplinary care opportunities [56,57] (for local adaptation), and synthesized literature sources [4,5,6,7,18,38,42,44,45,46,58,59]. ^e^ Across global settings, access to nutrition specialists such as Dietitians, Nutritionists, Medical Nutrition Specialists and suitably trained Nurse practitioners varies considerably. Whether access to specialist nutrition care is routinely available, and/or what the referral requirements are for specialist care, are highly dependent on local resources and care processes.

The SIMPLER Nutrition Pathway for Fragility Fractures is illustrated in Figure 1. SIMPLER has been adapted from the SIMPLE Approach with permission and acknowledgment from Queensland Health [21,22]. SIMPLER also includes concepts and constructs from numerous existing nutrition models, pathways, and processes [24,42,43,44,45,46,47]. The SIMPLER pathway is primarily designed to address protein-energy malnutrition and undernutrition in older adults. However, its adaptable framework allows it to be effectively applied elsewhere, for example, in secondary fracture prevention settings to identify and support individuals at risk of nutrition-related bone disease.

The following section provides a brief overview and rationale for the key components of the SIMPLER Pathway, as developed and refined through the co-creation and Knowledge-to-Action phases.

#### 3.3.1. S—Screening

The grey diamond box provides guidance on nutrition screening. This triages patients to the three categories of care (Figure 1). The SIMPLER Pathway prioritizes early, interdisciplinary, multicomponent “Supportive Nutrition Care” (Orange) for all hip fracture patients, regardless of nutrition risk score, level of adiposity, or usual intake. This helps teams to align care with evidence-based guidelines and standards, recognizing that while most at-risk older inpatients do not require specialist nutrition care, they do routinely require timely, multidisciplinary, multicomponent, ‘supportive’ nutrition actions [8,9,17,18,22]. This approach also recognizes the limited availability of timely access to nutrition specialists in many global settings [21,22,60]. Supportive nutritional care should continue until patients are assessed as well-nourished and not at risk of malnutrition, with adequate intake to meet post-operative, and post-trauma requirements.

A small proportion of hip fracture patients who require immediate specialist input should be referred for “Specialist Nutrition Care” in line with local referral processes (Red). Similarly, a small proportion of patients may be assessed as well nourished, not at risk of becoming malnourished, and already demonstrating adequate protein and energy intake to meet post-injury requirements. These patients and other fragility fracture patients screened as ‘not at risk’, should be triaged to “Standard Nutrition Care” (Green). Standard care should still provide high-quality, nutrient-dense food and fluid choices, therapeutic diets, mealtime support, and regular re-screening [17,47].

For other fragility fracture patients, the pathway supports local selection of validated screening tools (e.g., the Mini-Nutritional Assessment Short Form [49], Malnutrition Screening Tool [50], Canadian Nutrition Screening Tool [61], or Malnutrition Universal Screening Tool [45]). It also encourages teams to consider routinized identification and supportive care for other high-risk individuals (e.g., those with delirium or periprosthetic fractures). The pathway also facilitates nutrition rescreening and reassessment, ensuring appropriate transitions between standard (green), supportive (orange), and specialist (red) nutrition care as needed [21].

The examples provided above, and in the following sections, are not intended to be prescriptive. Teams implementing SIMPLER are encouraged to select, test, and adapt the tools, techniques, and care actions that best suit their local context and available resources. This approach ensures the SIMPLER model remains adaptable across diverse clinical and cultural settings, with emphasis on consistent delivery of the core components rather than uniform use of specific tools.

#### 3.3.2. I—Interdisciplinary Nutrition Assessment and Reassessment

Those screened as at risk should receive a thorough nutrition assessment. The rationale and opportunities for interdisciplinary nutrition assessment and reassessment processes are described elsewhere [2,7,14,17,18,47,62,63,64,65,66]. Unless specialist care is required, supportive nutrition care processes may be commenced prior to assessment.

#### 3.3.3. M—Make the Diagnosis, Inform the Patient/Carer, and Document

After nutrition assessment, a key action is to ensure that all those identified as at risk receive a nutrition diagnosis. This should be documented in the medical record. The most reported nutritional diagnosis in hip fracture is malnutrition (undernutrition, protein-energy malnutrition). The misclassification, under-identification, and under-documentation of malnutrition are common, particularly in those who are overweight or obese [15,67]. A diagnosis of malnutrition should be made by applying a validated diagnostic tool. Examples of these include but are not limited to, the Global Leadership Initiative on Malnutrition (GLIM) criteria [51], the International Classification of Diseases (ICD) criteria [52], the Subjective Global Assessment [53], and the Mini-Nutritional Assessment Short Form (score < 8) [49,54]. Examples of additional nutrition diagnoses commonly observed in clinical practice across hip fracture and other fragility fracture settings, mapped to the International Dietetics and Nutrition Terminology, are provided elsewhere [55,62,68,69,70].

To support informed consent and shared decision-making, individuals screened as at risk should be informed of their screening results and any subsequent assessments. This should include communicating any relevant diagnoses, which can also help improve adherence to nutrition interventions [71,72]. Unfortunately, in many settings, patients and/or their carers report being unaware of their risk status and/or nutrition diagnostic results [22,60]. Again, any diagnoses made should be documented in the treatment record.

As previously highlighted, SIMPLER is designed to be non-prescriptive with respect to assessment and diagnostic tools. What should be emphasized is the consistent diagnosis, provision of information, and documentation, while allowing flexibility in the choice of validated tools based on local context, workforce, and resources.

#### 3.3.4. P—Plan Supportive Interventions with the Patient

Care planning, including person-centered goal setting, is an essential component of fragility fracture care and rehabilitation [73]. Actively engaging individuals in their recovery can support care approaches that are in line with their personal values, needs, and functional priorities. This can increase engagement, motivation, and adherence to treatment, and ultimately improve patient and healthcare outcomes. In some situations, following shared decision-making and informed processes, patients and their care partners may actively and appropriately choose to focus on food and fluids for comfort, for example, where a focus is on care at the end of life. In many settings, processes to engage patients in care planning and goal-setting components are under-implemented [22,36,60,74,75].

#### 3.3.5. L—Implement Supportive Interventions

A range of nutrition interventions can be implemented at the individual, unit, hospital, or policy level [24,44,47]. As no single intervention effectively addresses malnutrition in hip fracture care, interdisciplinary, multicomponent approaches are recommended [22,23,55,57]. The SIMPLER Pathway and protocol provide a structured framework for teams to test, select, and implement targeted nutrition interventions for patients with or at risk of malnutrition who do not require specialized care. Priority core improvement opportunities, integrating evidence from primary research, systematic reviews, meta-analyses, clinical guidelines, and care standards, are presented as “The SIMPLER Six Improvement Opportunities” (Table 2). The tools and strategies referred to in Table 2 are provided as illustrative examples; local teams are encouraged to adapt or substitute alternative evidence-based approaches that are better suited to their clinical context, resources, and population needs. Local implementation teams may choose to prioritize additional or alternative evidence-based interventions.

#### 3.3.6. E—Evaluate Nutrition Care Provided

SIMPLER supports implementation teams to consider, test, and choose a small set of nutrition process measures to apply at baseline and post-implementation to (i) identify key areas for improvement, and (ii) identify whether changes made are likely to have led to an improvement. Additional measures within the scope of a quality improvement activity may be conducted during implementation to guide implementation and sustainment. Table 3 provides examples of core nutrition care process measures informed by primary research, systematic reviews and meta-analysis, evidence-based guidelines and care standards, and clinician consensus [6,8,17,18,22,39,40]. Examples are provided for audits, patient/carer reported measures, and treating clinician estimates. These examples are not intended to be prescriptive; they may be adapted, modified, or replaced with alternative measures that better align with local priorities, data availability, and quality improvement goals as part of the SIMPLER test-and-choose approach. Examples of implementation measures aligned with the RE-AIM (Reach, Effectiveness, Adoption, Implementation, Maintenance) framework [41] are provided in Supplement Materials S1 (Table S1: Example implementation measures aligned with the RE-AIM framework).

#### 3.3.7. R—Review, Reform, and Redesign Practice

The SIMPLE actions above are directed towards facilitating supportive nutrition care processes. The final key SIMPLER action is to review, reform, and redesign practice. Examples of opportunities for teams to consider when reviewing, reforming, or redesigning practice are outlined in Supplementary Materials (Table S2: Opportunities for teams to consider when reviewing, reforming or redesigning practice): Opportunities for teams to consider when reviewing, reforming, or redesigning practice).

### 3.4. The SIMPLER Protocol and Toolkit

“The SIMPLER Nutrition Pathway for Fragility Fractures—Implementation Protocol Template for Local Tailoring and Co-Creation” is a field-tested, co-created protocol template designed to support treating teams across diverse global settings in selecting, tailoring, and implementing SIMPLER. The protocol provides a structured approach to support sites in implementing SIMPLER, aligned with the model for improvement: forming teams, setting aims, selecting measures, testing interventions, and evaluating outcomes [28]. The Pathway, protocol, and toolkit resources are available on the Fragility Fracture Network website, www.fragilityfracturenetwork.org, or on request from the contact author.

## 4. Discussion

Across global settings, a major gap remains between nutritional recommendations and real-world practice for older adults with fragility fractures, particularly those with hip fractures [35,36,37,38]. This manuscript presents a novel, field-tested, expert-endorsed, and locally adaptable pathway that serves as a template for adopting sites to implement and evaluate nutrition improvements to close the evidence-to-practice gap.

A primary challenge to making nutrition care SIMPLER on a global scale is the diversity of contexts in which those with hip fractures and other fragility fractures are treated [87,88]. Consequently, the final pathway and implementation protocol avoids a prescriptive, algorithmic, ‘one size fits all’ approach, with a requirement for local sites to adapt to context, preferably applying principles of co-creation and/or similar processes [30,34,89,90,91]. For example, there are no gold standards for nutrition screening and diagnosis; recommending a specific screening or diagnostic tool was quickly identified as a key barrier to applying the tool in other settings where different tools were applied [15,16,92,93]. Similarly, requiring detailed assessment and intervention by specialists, for example, dietitians or medical nutrition experts, was considered unrealistic across many global settings where timely access to nutrition specialists is not available [22,38,94,95,96]. Attempts to define food, fluid, and oral nutrition supplement prescriptions and dosages that could be realistically offered across global contexts were also not considered realistic or feasible across a large-scale implementation program [39,40,97,98].

Implementing and evaluating change in complex healthcare systems is challenging [99,100]. Identifying the evidence-practice gap, reaching a consensus, and identifying new adopter sites ready to implement will not guarantee meaningful, scalable, or sustainable improvement [101,102]. The second key challenge is ‘how’ to implement [103,104,105,106]. SIMPLER teams must carefully unpack the ‘black box’ of implementation if improvements are likely to be sustained and spread [107,108]. This paper applies the KTA as a primary implementation model and the Model for Improvement for guiding quality improvement processes. However, across the course of implementation, numerous concepts and constructs from other theories, models, and frameworks were integrated across action–reflection cycles [101,109,110,111,112,113,114,115,116,117,118,119]. Given the proliferation of theories, models, and frameworks in the overlapping fields of implementation science, knowledge translation, and complexity science, adopting sites should engage implementation support practitioners or specialists, where available [106,120,121,122]. Sites should also consider joining networks of like-minded people or communities of practice, including those who have started their SIMPLER journey, for peer support [123,124].

A third key challenge is measurement [28,97,99,100,125]. The final core measures provided and approved were iteratively developed across many engagement processes and aligned to existing evidence. However, those listed in Table 3 are not prescriptive. Although acknowledged as ‘core’ measures, the pathway supports sites to select, test, and adapt measures where required, and are also free to choose from these or other measures depending on context. Sites should take care to avoid over-measurement and/or over-interpretation of findings. Those implementing SIMPLER should be mindful of the distinction between quality improvement or implementation outcomes and clinical, treatment, healthcare, or cost-effectiveness outcomes [100,126]. In most cases, for the selected improvement opportunities provided, research methods to demonstrate efficacy or effectiveness are not required, as these already have a strong evidence base. Instead, teams are encouraged to focus on mixed-methods data that can be collected during routine clinical care—data that are appropriate, feasible, and useful both for identifying improvement priorities and assessing whether changes have led to improvement [28,104].

A final challenge is the need to recognize national and international differences in the level of oversight required for quality assurance, improvement, and knowledge translation activities [127]. Confusion and differences may arise regarding when and where the SIMPLER implementation crosses over from quality improvement to research [128]. Others have observed that it can be unhelpful to try and clearly separate quality assurance or improvement processes from research, and these and similar processes exist on a continuum of activity that can evolve over time from one form to another [128]. Given the observed differences across settings, sites should ensure that prior to implementation, local quality and/or ethics approvals are in place to ensure adherence to, relevant governance, policy, professional, regulatory, and resource requirements.

This manuscript purposely reports the development and field-testing process, implementation fidelity, uptake, and sustainability of SIMPLER in a small number of sites. This is a key limitation. Future works will present implementation processes applied across globally diverse settings and present mixed methods implementation outcomes evaluation.

## 5. Conclusions

Malnutrition is one of the strongest predictors of poor outcomes after hip fracture. Despite strong evidence supporting change, there remains a gap between nutritional recommendations and practice. The SIMPLER Nutrition Pathway and Protocol for Fragility Fractures provides a novel opportunity to support local sites to co-create implementation efforts which can close this evidence-to-practice gap. with strong demand for adoption already demonstrated across diverse global settings, we invite you to consider tailoring SIMPLER to your setting and evaluating improvement efforts to bridge the evidence-to-practice gap.

## Figures and Tables

**Table 1:** SIMPLER co-creation engagement processes.

Process	Region: n
Semi-structured healthcare professional interviews (number of interview sets)	Global: 2
Interprofessional workshops (number of workshops)	Global: 2 Asia Pacific: 4 Europe: 3 North America: 1
Interprofessional focus group meetings (number of meetings)	Global: 4 North America: 1 Europe: 3 Asia-Pacific: 1
Site support meetings (number of meetings)	Asia Pacific: 6 Europe: 14 South America: 2
Webinars (number of webinars)	Asia Pacific: 3 Europe: 3
Plenary/keynote/symposia/platform presentations (number of presentations)	Global: 4 Asia-Pacific 10 Europe: 12 North America: 2 South America: 1
Web-based surveys, polls, and consensus processes (number conducted)	Global: 5 Asia Pacific: 1
Patient level audits	Asia Pacific: 2
YouTubes, PodCasts	Global:6 AsiaPacific: 7
Web-based and face-to-face Education Modules and Lectures (number conducted)	Global: 1 Europe: 1 Asia-Pacific: 1
Strategic meeting presentations (FFN Executive/Board, Regionalisation, Education, Scientific Committees; Special Interest Advisory Boards (number of presentations)	Global: 6 Europe: 4 Asia-Pacific: 3

SIMPLER co-creation engagement processes across global, regional, and hospital levels. These included qualitative interviews, focus groups, workshops, and web-based surveys to inform the development and refinement of the SIMPLER Pathway and Toolkit. All processes were novel. Surveys and consensus activities were routinely pretested with input from interdisciplinary experts to ensure face and content validity. Interviews and focus group discussions were also underpinned by established evidence and implementation science and quality improvement models, supporting construct validity. Published examples are already available [35,36,37,38] with additional manuscripts and audit reports currently in preparation for peer review.

**Table 2:** Evidence-informed SIMPLER Nutrition Improvement Opportunities (The SIMPLER Six).

Improvement Opportunities	Evidence-Informed Rationale
1. Avoid unnecessary, prolonged, or repeated fasting	Unnecessary, prolonged, or repeated fasting is harmful and should be avoided [8,9,18,39,40,76].
2. Offer information about nutrition [risk] status	Up to one in two hip fracture patients are malnourished on admission to the hospital; hip fracture patients rarely meet post-operative nutritional requirements in the absence of early, interdisciplinary, multicomponent interventions [8,11,17,18,23,55]. Evidence suggests malnutrition screening tools have limited criterion validity in hip fracture; therefore, all hip fracture patients should be treated as ‘at risk’ of malnutrition, and offered information about their nutrition risk status, until a systematic nutrition assessment is performed by a trained person [8,9,15,17,18,39,40,77]. This assessment should apply a tool validated for the purposes of diagnosing protein/energy malnutrition, as well as identifying ongoing nutrition risk factors, for example, inadequate low intake, high requirements, or nutrient availability issues [49,51,53,78]. Following assessment, patients (or carers where appropriate) should be offered diagnostic advice regarding whether they are malnourished or remain at risk of malnutrition [17,18,79].
3. Offer information about nutrition interventions ^a^	Interdisciplinary, multicomponent interventions should be offered to all hip fracture patients unless assessed as ‘not at risk’ or not in line with patient treatment preferences [8,9,17,18,39,40,80]. This should include the provision of information or education to support informed consent, shared decision-making regarding treatment choices, and adherence to interdisciplinary, multicomponent interventions [79,80].
4. Offer high-quality, high-protein/energy food and fluids, with regular intake assessment ^a^	All hip fracture inpatients, unless assessed as well-nourished and not at risk of malnutrition, should be offered high quality, appropriately textured, high protein/energy food and fluids, fortified food, additional snacks, and/or finger foods to support adequate dietary intake [7,8,9,17,18,23,55]. Consumption of these should be assessed to support corresponding adjustment of interventions [17,18].
5. Offer oral nutritional supplements, * with regular intake assessment ^a^	All hip fracture inpatients, unless assessed as well-nourished and not at risk of malnutrition, should be offered oral nutritional supplements, in combination with dietary information/counselling and food fortification, to improve patient and healthcare outcomes [4,5,6,17,18]. Intake of these should be regularly assessed [17,18].
6. Offer malnutrition [risk] status and treatment plan to be provided to the preferred post-hospital healthcare provider ^b^	Ongoing nutrition care should be offered to all inpatients who remain at risk of malnutrition or are malnourished at the time of discharge from the hospital [17,18]. Where consent is provided, a referral should be made to the patient’s preferred healthcare provider, which includes their nutrition status and treatment plan [80].

Evidence strength varies across SIMPLER actions and reflects a combination of systematic reviews, expert consensus, and guideline recommendations. ^a^ Within 72 h of presentation (target 24 h) with a hip fracture; unless already assessed well-nourished/not at risk. ^b^ On or after discharge from the hospital; unless assessed well-nourished/not at risk. * Oral nutrition supplements are defined as protein and energy nutrient-dense products purposed to increase dietary intake when diet alone is likely to be inadequate to meet nutritional requirements. These may include energy and protein-enriched drinks (e.g., milk, soy, protein-fortified juice flavours), powders, soups, and/or desserts [81].

**Table 3:** Example SIMPLER core nutrition care measures for hip fracture patients.

Core Measure	Audit Source: Medical Record, Bed Chart, and/or Discharge Documentation	Patient/Carer ^c^ Reported Measure Source: Standardized PREM Collected by Designated Person	Treating Clinician ^d^ Estimate Example Source: Clinician Survey (Paper and/or Electronic Versions)
Unnecessary, prolonged, or repeated fasting? [8,9,17,18,37,76,82,83,84]	Fasted for more than about 6 h before surgery, or fasted more than once? (No; Yes, or not documented)	Were you fasting for more than about 6 h before your surgery, or fasted more than once? (No; Yes, or not documented)	What percentage of all hip fracture patients you have cared for in the past month are fasted for more than about 6 h before surgery, or fasted more than once? 0–25|25–50|50–75|75–100
Awareness of nutrition [risk] status? ^a^ [7,8,9,17,18,22,24,39,40,44,80]	Documented nutrition assessment *and* provision of malnutrition [risk] assessment to patient/caregiver? ^a^ (Yes; No, or not documented)	Anybody who is 65 or older who has had hip fracture surgery should have a nutritional assessment. Have you been provided with the results of your nutritional assessment? (Yes; No, or don’t know)	What percentage of all hip fracture patients you have cared for in the past month have had a nutrition [risk] assessment *and* are aware of their nutrition [risk] status? 0–25|25–50|50–75|75–100
Provided with information/education about nutrition? ^a^ [7,8,17,18,22,24,39,40,44,57,58,59,85]	Documented provision of information/education about nutrition? ^a^ (Yes; No, or not documented)	Have you received any information or education about nutrition since you have been in the hospital? (Yes; No, or don’t know)	What percentage of all hip fracture patients you have cared for in the past month are provided with information/education about nutrition? 0–25|25–50|50–75|75–100
Provided high protein/energy food and fluids *and* intake is regularly assessed? ^a^ [17,18,22,39,40,58,85]	Documented evidence of provision of high protein/energy food and fluid choices *and* assessment of food and fluid intake? ^a^ (Yes; No, or not documented)	Are you receiving high-quality, high-protein food and fluid choices, *and* has anybody asked you how much you have been eating? (Yes; No, or don’t know)	What percentage of all hip fracture patients you have cared for in the past month have received high protein/energy foods *and* have had their intake assessed within 72 h of surgery? 0–25|25–50|50–75|75–100
Provided with oral nutritional supplements * *and* intake is regularly assessed? ^a^ [4,5,6,17,18,39,40,58,85,86]	Documented evidence of the provision of supplements *and* assessment of supplement intake? ^a^ (Yes; No, or not documented)	Are you receiving oral nutrition supplements, *and* have you been asked about your intake of these? (Yes; No, or don’t know)	What percentage of all hip fracture inpatients you have cared for in the past month have been provided oral nutritional supplements *and* have had their intake of these assessed within 72 h of surgery and weekly thereafter? 0–25|25–50|50–75|75–100
Malnutrition [risk] status and nutrition plan provided to a post-hospital healthcare professional? ^b^ [8,17,18,22,39,40,80]	Evidence of malnutrition [risk] status *and* nutrition plan in hospital discharge summary/discharge letter or other discharge documentation? (Yes; No, or not documented)	Has anybody asked you if they can give your nutrition diagnosis and plan to your preferred post-hospital healthcare provider? (Yes; No, or don’t know)	What percentage of malnourished (or still at risk) hip fracture patients have had their malnutrition (risk) status documented in their medical discharge summary *and* have a nutrition treatment plan included in their discharge paperwork? 0–25|25–50|50–75|75–100

^a^ Within 72 h of presentation with a hip fracture and weekly thereafter; unless already assessed well-nourished/not at risk. ^b^ On or after discharge from the hospital; unless assessed well-nourished/not at risk. ^c^ If the patient is unable to answer, ask the carer or treating nurse. ^d^ Completed by interprofessional team members. * Oral nutrition supplements are defined as protein and energy nutrient-dense products intended to increase dietary intake when diet alone is likely to be inadequate to meet nutritional requirements. These may include energy and protein-enriched drinks (e.g., milk, soy, protein-fortified juice flavours), powders, soups, and/or desserts.

## Data Availability

Original site-specific data underpinning the aggregated findings presented in this article are not readily available because this manuscript is presented within the scope of a quality improvement project. Qualitative findings are part of an ongoing study planned for publication elsewhere. Requests to access the datasets should be directed to corresponding author.

## Supplementary Materials

The following supporting information can be downloaded at https://www.mdpi.com/article/10.3390/nu17121987/s1, Table S1: Example implementation measures aligned with the RE-AIM framework; Table S2: Opportunities for teams to consider when reviewing, reforming or redesigning practice.

## Abbreviations

The following abbreviations are used in this manuscript:

FFN	Fragility Fracture Network
KTA	Knowledge-To-Action (KTA)
PDSA	Plan–Do–Study–Act
RE-AIM	Reach, Effectiveness, Adoption, Implementation, Maintenance
