# Supplementary material for: The SIMPLER Nutrition Pathway for Fragility Fractures: A Quality Improvement Initiative
Source: Nutrients. 2025 Jun 12;17(12):1987. doi: 10.3390/nu17121987 (PMC12195995; doi:10.3390/nu17121987)
Supplement: Supplementary file 1 [file nutrients-17-01987-s001.zip › nutrients-3666564-supplementary.pdf]

nutrients

MDPI

Table S1: Example implementation measures aligned with the RE-AIM framework [1]

RE-AIM	Measure and examples
Reach: The number, proportion, and/or representativeness of participating inpatients	Patient level audit example: The total number, age and sex of included patients as a proportion of total target population.<br>Clinician survey example: Estimated total number, proportion, and representativeness of included patients following implementation commencement.<br>Qualitative example: strong themes of high-level engagement of individuals with exemplar quotes.
Effectiveness: The impact of SIMPLER implementation on important outcomes, for example care delivered in line with evidence-based guidelines or standards	Patient level audit example: The pre- and post-proportions of hip fracture inpatients who received two or more SIMPLER core supportive nutrition care interventions.<br>Clinician estimate example: Estimated pre- and post-proportions of hip fracture inpatients who received two or more SIMPLER core supportive nutrition care interventions.<br>Qualitative example: strong themes of implementation influence on alignment of care to evidence based practice or patient experiences, with exemplar quotes.
Adoption: The total count, percentage, or inclusiveness of staff, settings, systems, and intervention agents (those delivering the program) who are willing to start the program	Staff level example: percent of invited staff that participated.<br>Clinician survey example: Estimated total number, proportion, and representativeness of staff willing to participate in SIMPLER implementation.<br>Qualitative example: strong themes highlighting why settings or staff did, or did not participate in the SIMPLER program, with exemplar quotes.
Implementation: Was SIMPLER implementation done as planned, on time, and within budget?	Clinical survey level example: changes in the pathway, core measures, core actions, resource requirements or costs.<br>Qualitative example: Strong themes highlighting barriers and enablers to implementation as planned.
Maintenance: How much has SIMPLER become a regular part of organizational practice and policies? How well has the program's effects lasted six months or more?	Patient level audit example: Repeat above effectiveness measure 6 months after last formal engagement with the facilitators?<br>Setting level example: Are there examples of impact on policy, procedures, or strategy?<br>Qualitative example: Case study, patient or staff stories, or strong themes with exemplar quotes providing examples of where the implementation has been sustained, or routinised into practice and policy.

1. Glasgow, R.E.; Harden, S.M.; Gaglio, B.; Rabin, B.; Smith, M.L.; Porter, G.C.; Ory, M.G.; Estabrooks, P.A. RE-AIM Planning and Evaluation Framework: Adapting to New Science and Practice With a 20-Year Review. *Front Public Health* **2019**, *7*, 64-64, doi:10.3389/fpubh.2019.00064.

Table S2: Opportunities for teams to consider when reviewing, reforming or redesigning practice.

**Opportunities**	**Examples**
Audits	Australian and New Zealand Hip Fracture Registry[1]<br>ANZHFR Nutrition Sprint Audit [2]<br>ANZHFR Preoperative Fasting Sprint Audit[3]<br>FFN Minimum Common Dataset[4]<br>Scottish Hip Fracture Registry Audit[5]<br>SIMPLER Measures[6]
Clinical systems, governance and organisational processes	Comprehensive Care Committees [7]<br>Foodservice systems / Care Catering / Hospital Catering [8-10]<br>Integrated Electronic Medical Records / Meal Ordering Systems [11]<br>Nutrition steering committees and support Teams [8]
Evidence based recommendations, guidelines and care standards	Australian Hip Fracture Clinical Care Standards[12]<br>ESPEN guidelines[13-15]<br>Scottish Standards of Care For Hip Fracture Patients [16]<br>Canadian Malnutrition Prevention, Detection and Treatment standard [17]
Models or frameworks that support integrated care	Integrated Pathway for Acute Care [18]<br>Orthogeriatric framework[19]<br>Primary care nutrition pathway [20]<br>SIMPLE / SIMPLER Models[21,22]<br>Model for improving nutrition care[23]<br>The Sustain and Spread Framework[24]
Patient and healthcare team stories and person reported measures	ANZHFR Reports [1]<br>My Hip My Voice [25]<br>Patient Stories Systematic Review [26]
Workforce capacity, capability and role optimization	Nurse Opportunities (Sytematic Review)[27]<br>Allied Health Assistant Opportunities (Systematic Review)[28-30]<br>Nutrition education and professional development opportunities

1. Australian and New Zealand Hip Fracture Registry. ANZHFR Website. Available online: https://anzhfr.org/ (accessed on 23 January, 2025).
2. ANZ Hip Fracture Registry. ANZ HFR Nutrition Sprint Audit. Available online: https://anzhfr.org/sprintaudits/ (accessed on
3. Australian and New Zealand Hip Fracture Registry. Preoperative Fasting Sprint Audit. Available online: https://anzhfr.org/sprintaudits/ (accessed on January 23, 2025).
4. Fragility Fracture Network. Minimum Common Dataset. Available online: www.fragilityfracturenetwork.org (accessed on
5. Scottish Hip Fracture Audit. Scottish Hip Fracture Audit Reports. Available online: https://www.shfa.scot.nhs.uk/Reports/index.html (accessed on 25 January, 2024).
6. Fragility Fracture Network SIMPLER Implementation Steering Committee. SIMPLER Nutrition Measures and Actions recommended for implementation - consensus from the Fragility Fracture Network (Global) Implementation Steering Committee. **2024**.

7. Australian Commission on Safety and Quality in Healthcare. Comprehensive Care Standard. Available online: https://www.safetyandquality.gov.au/standards/nsqhs-standards/comprehensive-care-standard#background-to-this-standard (accessed on 23 January 2025).
8. Cederholm, T.; Barazzoni, R.; Austin, P.; Ballmer, P.; Biolo, G.; Bischoff, S.C.; Compher, C.; Correia, I.; Higashiguchi, T.; Holst, M.; et al. ESPEN guidelines on definitions and terminology of clinical nutrition. *Clinical Nutrition* **2017**, *36*, 49-64, doi:10.1016/j.clnu.2016.09.004.
9. Young, C.; Farrah, K. *Room service food delivery models for hospital in-patients: A review of clinical effectiveness, cost-effectiveness, and guidelines.* ; Canadian Agency for Drugs and Technologies in Health: June 17 2019.
10. Neaves, B.; Bell, J.J.; McCray, S. Impact of room service on nutritional intake, plate and production waste, meal quality and patient satisfaction and meal costs: A single site pre-post evaluation. *Nutrition & Dietetics* **2022**, *79*, 187-196, doi:https://doi.org/10.1111/1747-0080.12705.
11. Prgomet, M.; Li, J.; Li, L.; Georgiou, A.; Westbrook, J.I. The impact of electronic meal ordering systems on hospital and patient outcomes: A systematic review. *International Journal of Medical Informatics* **2019**, *129*, 275-284, doi:https://doi.org/10.1016/j.ijmedinf.2019.06.023.
12. Australian Commission on Safety and Quality in Healthcare. Hip Fracture Clinical Care Standard. Available online: https://www.safetyandquality.gov.au/standards/clinical-care-standards/hip-fracture-care-clinical-care-standard (accessed on 23 January, 2025).
13. Volkert, D.; Beck, A.M.; Cederholm, T.; Cruz-Jentoft, A.; Goisser, S.; Hooper, L.; Kiesswetter, E.; Maggio, M.; Raynaud-Simon, A.; Sieber, C.C.; et al. ESPEN guideline on clinical nutrition and hydration in geriatrics. *Clinical Nutrition* **2019**, *38*, 10-47, doi:10.1016/j.clnu.2018.05.024.
14. Volkert, D.; Beck, A.M.; Cederholm, T.; Cruz-Jentoft, A.; Hooper, L.; Kiesswetter, E.; Maggio, M.; Raynaud-Simon, A.; Sieber, C.; Sobotka, L.; et al. ESPEN practical guideline: Clinical nutrition and hydration in geriatrics. *Clinical nutrition (Edinburgh, Scotland)* **2022**, *41*, 958-989, doi:10.1016/j.clnu.2022.01.024.
15. Druml, C.; Ballmer, P.E.; Druml, W.; Oehmichen, F.; Shenkin, A.; Singer, P.; Soeters, P.; Weimann, A.; Bischoff, S.C. ESPEN guideline on ethical aspects of artificial nutrition and hydration. *Clinical nutrition (Edinburgh, Scotland)* **2016**, *35*, 545-556, doi:10.1016/j.clnu.2016.02.006.
16. Scottish Government. Scottish standards of care for hip fracture patients. **2024**.
17. Health Standards Organisation. CAN/HSO 5066:2021 - Malnutrition Prevention, Detection, and Treatment. Available online: https://healthstandards.org/standard/malnutrition-prevention-detection-and-treatment/ (accessed on 17 February 2025).
18. Keller, H.; Laur, C.; Atkins, M.; Bernier, P.; Butterworth, D.; Davidson, B.; Hotson, B.; Nasser, R.; Laporte, M.; Marcell, C. Update on the Integrated Nutrition Pathway for Acute Care (INPAC): post implementation tailoring and toolkit to support practice improvements. *Nutrition journal* **2018**, *17*, 1-6.
19. Network, F.F. Fragility Fracture Network Orthogeriatric Framework. Available online: https://fragilityfracturenetwork.org/ffn-resources/ (accessed on 12 September).
20. Canadian Malnutrition Task Force. Primary Care Nutrition Pathway for Hospital to Community Transitions. Available online: https://nutritioncareincanada.ca/sites/default/uploads/files/Pathways/Nutrition_Care_Pathway_for_Primary_Care_2020.pdf (accessed on 17 February, 2025).
21. Bell, J.J.; Young, A.; Hill, J.; Banks, M.; Comans, T.; Barnes, R.; Keller, H.H. Rationale and developmental methodology for the SIMPLE approach: A Systematised, Interdisciplinary Malnutrition Pathway for impLementation and Evaluation in hospitals. *Nutrition & dietetics: the journal of the Dietitians Association of Australia* **2018**, *75*, 226-234, doi:10.1111/1747-0080.12406.

22. Bell, J.J.; Young, A.M.; Hill, J.M.; Banks, M.D.; Comans, T.A.; Barnes, R.; Keller, H.H. Systematised, Interdisciplinary Malnutrition Program for impLementation and Evaluation delivers improved hospital nutrition care processes and patient reported experiences – An implementation study. *Nutrition & Dietetics* **2021**, *78*, 466-475, doi:https://doi.org/10.1111/1747-0080.12663.
23. Laur, C.; Valaitis, R.; Bell, J.; Keller, H. Changing nutrition care practices in hospital: a thematic analysis of hospital staff perspectives. *BMC Health Serv Res* **2017**, *17*, 498, doi:10.1186/s12913-017-2409-7.
24. Laur, C.; Bell, J.; Valaitis, R.; Ray, S.; Keller, H. The Sustain and Spread Framework: strategies for sustaining and spreading nutrition care improvements in acute care based on thematic analysis from the More-2-Eat study. *BMC health services research* **2018**, *18*, 1-11.
25. Australian and New Zealand Hip Fracture Registry. My Hip My Voice Resources. Available online: https://anzhfr.org/resourcesforpatients/ (accessed on 17 February, 2025).
26. Quah, E.L.Y.; Chua, K.Z.Y.; Lin, C.K.R.; Vijayan, A.V.; Abdul Hamid, N.A.B.; Owyong, J.L.J.; Satku, N.; Woong, N.; Lim, C.; Phua, G.L.G.; et al. The role of patients' stories in medicine: a systematic scoping review. *BMC Palliat Care* **2023**, *22*, 199, doi:10.1186/s12904-023-01319-w.
27. ten Cate, D.; Ettema, R.G.A.; Huisman-de Waal, G.; Bell, J.J.; Verbrugge, R.; Schoonhoven, L.; Schuurmans, M.J.; Group, t.B.C.R. Interventions to prevent and treat malnutrition in older adults to be carried out by nurses: A systematic review. *Journal of Clinical Nursing* **2020**, *29*, 1883-1902, doi:10.1111/jocn.15153.
28. Rushton, A.; Edwards, A.; Bauer, J.; Bell, J.J. Dietitian assistant opportunities within the nutrition care process for patients with or at risk of malnutrition: a systematic review. *Nutrition & Dietetics* **2021**, *78*, 69-85, doi:10.1111/1747-0080.12651.
29. Rushton, A.; Young, A.; Keller, H.; Bauer, J.; Bell, J. Delegation Opportunities for Malnutrition Care Activities to Dietitian Assistants—Findings of a Multi-Site Survey. *Healthcare* **2021**, *9*, 446.
30. Rushton, A.; Bauer, J.; Young, A.; Keller, H.; Bell, J. Barriers and Enablers to Delegating Malnutrition Care Activities to Dietitian Assistants. *Nutrients* **2022**, *14*, doi:10.3390/nu14051037.
